# Author’s Reply: Examining Multimodal AI Resources in Medical Education: The Role of Immersion, Motivation, and Fidelity in AI Narrative Learning

**DOI:** 10.2196/72336

**Published:** 2025-03-18

**Authors:** Tyler Bland

**Affiliations:** 1 Department of Medical Education, University of Idaho, 875 Perimeter Drive MS 4061, Moscow, ID, 83844-9803, United States, 1 5092090908

**Keywords:** artificial intelligence, cinematic clinical narrative, cinemeducation, medical education, narrative learning, pharmacology, AI, medical students, preclinical education, long-term retention, AI tools, GPT-4, image, applicability, CCN

I extend my sincere appreciation for the thoughtful critique [1] of my study, “Enhancing Medical Student Engagement Through Cinematic Clinical Narratives: Multimodal Generative AI–Based Mixed Methods Study” [2]. The author’s insights regarding engagement mechanisms, theoretical expansion, and methodological refinements offer valuable perspectives that contribute to the broader discourse on the pedagogical applications of generative artificial intelligence in medical education.

While the Cognitive Affective Model of Immersive Learning framework originated to explain learning with immersive virtual reality technologies [3], I concur that its underlying principles are applicable to my study. The debate over the role of media versus instructional methods in learning has been longstanding. While some argue that the medium itself shapes cognition, social structures, and cultural norms [4], others reject this notion, asserting that media are merely delivery mechanisms and that instructional methods alone drive learning outcomes [5]. The Cognitive Affective Model of Immersive Learning reframes this debate by emphasizing that it is not the medium (eg, immersive virtual reality) that inherently enhances learning, but rather how instructional methods leverage the unique affordances of that medium. In the context of cinematic clinical narratives (CCNs), the structured narrative and multimodal design capitalize on engagement mechanisms similar to those observed in immersive learning. Future research could further examine how instructional design within CCNs optimally harnesses these principles to promote knowledge retention and clinical application.

The author’s recommendation of the integration of pretest and posttest methodologies is well-founded. While the published study employed posttest assessments to measure comprehension, incorporating pretest measures would facilitate a more granular evaluation of baseline knowledge and attitudinal shifts attributable to CCNs. Furthermore, longitudinal assessments could provide critical insights into the durability of knowledge retention and the sustained impact of CCNs over extended timeframes. I aim to incorporate these into future studies.

The author’s call for broader contextual applications of CCNs beyond traditional classroom settings is well-taken. While the study examined CCN implementation within a structured learning environment, I am currently working on converting CCNs into self-contained short films that can be viewed online for self-directed learning. This adaptation aims to provide learners with greater flexibility while maintaining the engagement and narrative-driven structure of CCNs. Investigating how these self-contained films perform across varied instructional modalities could yield valuable insights into their scalability and applicability within diverse educational contexts.

Finally, I concur with the author’s observation that medical students are increasingly turning to digital platforms such as social media for information and engagement. Medical educators should take note and examine the factors that make these platforms so compelling. By understanding the draw of these digital environments, educators can incorporate similar characteristics into medical school learning materials to meet students where they are. Expanding CCN research to explore how elements such as interactivity, brevity, and personalization influence learner engagement could provide valuable insights into modernizing medical education. I am grateful for the astute observations and constructive recommendations by the author. These perspectives will undoubtedly inform my future research directions and further the integration of artificial intelligence–driven methodologies in my studies on medical education.

## Abbreviations

CCN: cinematic clinical narrative
